# Computational screening of antiviral candidates for Monkeypox virus DNA polymerase and A42R protein

**DOI:** 10.1371/journal.pntd.0013312

**Published:** 2025-07-29

**Authors:** Muhammad Amjid, Muhammad Maroof Khan, Stephen F. Pastore, John B. Vincent, Tahir Muhammad

**Affiliations:** 1 College of Pharmacy and Research Institute of Pharmaceutical Sciences, Gyeongsang National University, Jinju, Republic of Korea; 2 Department of Chemistry, Kohat University of Science and Technology, Kohat Khyber Pakhtunkhwa, Pakistan; 3 Department of Biotechnology, Agriculture University of Peshawar, Peshawar, Pakistan; 4 Molecular Neuropsychiatry & Development (MiND) Lab, Campbell Family Mental Health Research Institute, Centre for Addiction and Mental Health, Toronto, Ontario, Canada; 5 Institute of Medical Science, University of Toronto, Toronto, Ontario, Canada; 6 Department of Psychiatry, University of Toronto, Toronto, Ontario, Canada; Public Health Agency of Canada, CANADA

## Abstract

Monkeypox virus (MPXV) is emerging as a major concern in the field of infectious diseases. Current treatments are limited, highlighting the need for new therapeutic options. The use of computational methods, such as molecular docking and molecular dynamic (MD) simulations, is a valuable approach in identifying potential compounds that can target specific proteins of the virus, like the DNA polymerase and profilin-like protein profilin-like protein A42R (A42R) in this case, with the aim of controlling the disease. Our study focused on screening various libraries of compounds for predicted binding to MPXV DNA Polymerase (DPol) and A42R proteins, with the top-performing molecules identified based on their docking scores. Among these, Dorsilurin K and Mangostin in complex with DPol, whereas [2-oxo-2-[3-(3,4,5,6-tetrahydro-2H-azepin-7-ylsulfamoyl)anilino]ethyl] 3,5-dimethylbenzoate and N-[4-[2-[4-(4-methylphenyl)sulfonylpiperazin-1-yl]-2-oxoethoxy]phenyl]furan-2-carboxamide in complex with A42R stand out with notably high docking scores, suggesting they may have a good affinity for binding to the DPol and A42R proteins of MPXV respectively. MD simulations confirmed the stability of these ligand-protein complexes followed by evaluation of the ADMET and oral bioavailability analysis. However, it is important that computational methods can suggest promising candidates, *in vitro* and eventually *in vivo* studies are essential to validate these therapeutic candidates. Further studies on these compounds will provide insights into their efficacy, safety, and potential side effects. In conclusion, this study offers promising avenues for developing potential treatments for MPXV. If the identified compounds prove effective in further studies, it could be a significant breakthrough in managing this zoonotic disease.

## Author summary

The study focuses on finding antiviral drug candidates targeting the DNA Polymerase (DPol) and Profilin-like Protein A42R (A42R) of the Monkeypox virus (MPXV) using an *in-silico* approach. Various compound libraries were screened for potential inhibitors and the top-performing ligands were selected based on their predicted binding affinities. This study identified several ligands, including Dorsilurin K, Mangostin, dimethylbenzoate derivative and others that demonstrated strongly predicted binding to DPol and A42R, suggesting their potential as effective inhibitors of MPXV. The molecular dynamics simulations confirmed the stability of these ligand-protein complexes and further ADMET analysis indicated favorable pharmacokinetic properties. These findings provide a promising foundation for developing new antiviral treatments against MPXV, however further *in vitro* and *in vivo* studies are required to validate these results.

## Introduction

Monkeypox virus (MPXV), a member of the Poxviridae family, causes monkeypox disease and shares genetic similarities with other orthopoxviruses such as variola and vaccinia [1,2]. The MPXV genome can be divided into three segments: a core region, a left arm, and a right arm. Unlike orthopoxviruses that encode approximately 200 genes, MPXV carries roughly 190 genes. The core region is relatively conserved in the genome and encodes genes related to viral replication and assembly [3].

Although less lethal than smallpox, MPXV remains a significant health concern due to its high prevalence and potential for causing disability and disfigurement [4]. Clinical symptoms include malaise, severe headaches, fever with temperatures ranging from 38.5°C to 40.5°C [5], and the development of lesions on the face, extremities, oral mucous membranes, genitalia, conjunctivae, and cornea [6]. Additional symptoms encompass chills, weakness, fatigue, myalgia, lymphadenopathies in cervical, inguinal, and axillary regions, as well as ulcers and vesicles in the genital and anal areas [7]. In severe cases, complications such as pneumonia and sepsis can occur, carrying a high risk of mortality [8]. The incubation period ranges from 7 to 17 days, with fever subsiding three days after the rash onset. Lesions are characterized as painful, indurated, and edematous. Lymphadenopathy, which is absent in smallpox, triggers a robust immune response in monkeypox disease, distinguishing the two diseases. MPXV has an associated mortality rate of up to 10%, with a particular severity observed in pediatric patients [4]. MPXV can be transmitted through zoonosis and human-to-human contact. Modes of acquisition include percutaneous exposure, direct contact with skin (especially damaged skin), direct contact with mucous membranes (e.g., oral, vaginal, rectal), and inhalation of infected respiratory particles [4]. Potential sources of infection encompass infected individuals, animals, or contaminated objects. Notably, direct sexual contact has emerged as a primary mode of transmission in ongoing outbreaks, as indicated by the predominance of anogenital lesions in cases [9].

On August 14, 2024, the World Health Organization (WHO) declared the MPXV outbreak a public health emergency of international concern due to the rapid increase in infections [10].

Currently, there are no FDA-approved treatments for monkeypox disease. However, during the 2022 outbreak emergency, Tecovirimat received FDA approval as the sole antiviral medication to inhibit MPXV replication and manage severe symptoms [5,11]. Additionally, while smallpox medications such as Cidofovir, Brincidofovir, and Tecovirimat have been approved and the smallpox vaccine demonstrates 85% effectiveness against MPXV [5,12,13], it is important to clarify that these treatments also exert their effects against MPXV. Tecovirimat is the sole anti-poxvirus medication officially recommended by the US FDA [12,13]. While Cidofovir and Brincidofovir inhibit DNA replication and exhibit efficacy against numerous double-stranded DNA viruses [14,15], Tecovirimat displays a higher specificity for orthopoxviruses, preventing the formation of enveloped virions through inhibition of the conserved protein F13L [16].

In the present study, we employ an *in-silico* structure-based drug design approach to identify potential lead compounds from six distinct libraries, i.e., MedChemExpress NaturalProduct library [17], MPD3 medicinal plant database [18], alkaloid compound library [19], MedChemExpress flavonoid compound library [20], Enamine antiviral compound library [15], and express-pick library [19] targeting the DPol and A42R proteins of MPXV. We selected DPol and A42R proteins based on the previously reported studies using these to be the most important targets to screen drugs for MPXV. We conducted virtual screening of libraries by performing the molecular docking and subsequently subject the top ten hits to molecular dynamics simulations. These top hit compounds consist of DPol-MPD3_1, DPol - NaturalProduct_1, DPol-Express Pic_1, DPol-NaturalProduct_2, DPol-ExpressPick_2, A42R-ExpressPick_1, A42R-ExpressPick_2, A42R-ExpressPick_3, A42R-Enamine_1, and A42R-NaturalProduct_1.

**Fig 1:**
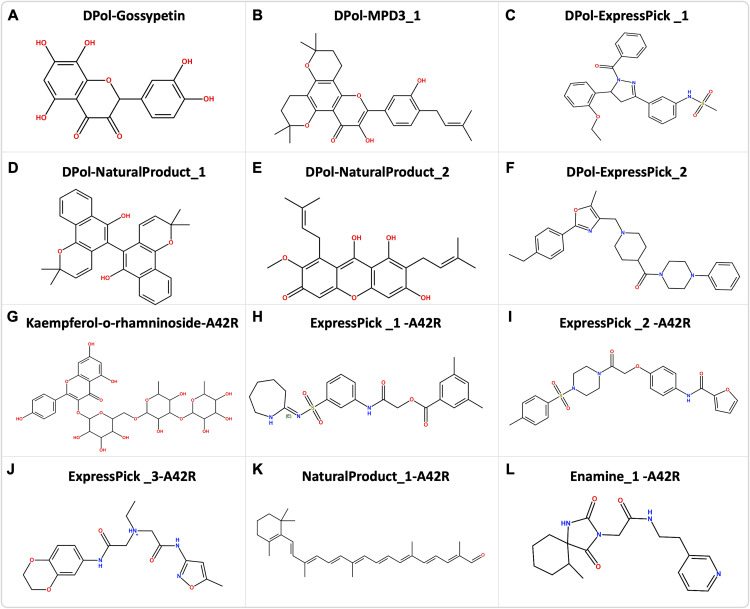
Structure of the hit compounds: Chemical structure of the 10 hit compounds identified in this study to have potentials antiviral activities against monkeypox virus (MPXV). Naming is based on the ranking of docking score from the compound library with the MPXV proteins (i.e. Compound’s library position in the docking score-MPXV protein).

We hypothesize that binding of specific small-molecule ligands to the active or allosteric sites of DPol and profilin-like protein A42R will inhibit their enzymatic or structural functions, respectively, thereby reducing viral replication and host cell infection. The chemical structures are given in Fig 1, and we will discuss these in detail below. Molecular docking and dynamic simulation analyses predict stable binding interactions of these compounds at the DPol and A42R binding sites. Finally, we performed the absorption, distribution, metabolism, excretion and toxicity (ADMET), and oral bioavailibity of the top hit ligands.

## Methods

### Compound library selection and preparation

Six distinct compound libraries were chosen for analysis, consisting of a Natural Product library, MPD3 medicinal plants database, flavonoid compound library (MedChemExpress), alkaloid compound library (Selleckchem.com), Enamine antiviral library, and express-pick library [15,17–20]. These libraries comprised 3948, 2295, 241, 401, 3200 and 3010 compounds, respectively, and were obtained in 3D Structure-data file (SDF) format. To ensure the pharmacokinetic viability of the compounds, an initial filter was applied based on Lipinski’s Rule of Five [21]. This selection filter helps in the inclusion and exclusion criteria for drug like compounds, for example, size of the compounds, polarity, number of bonds it makes and the distribution. These properties include that ligands should have no more than 5 hydrogen bond donors, no more than 10 hydrogen bond acceptors, a molecular mass less than 500 Da, polar surface area of less than 140Å, LogP > 5 (octanol-water coefficient, indicating better absorption). We used MOE’s in-built descriptor tool, arranged compounds in descending order for each descriptor and deleted those ligands that were violating the Lipinski rule. After this step, out of 13101 in total, 8564 compounds followed this criterion, and were prepared for subsequent molecular docking processes (Table 1).

**Table 1:** Compound libraries, number of total compounds, format of the library and their retrieval date.

Compound library	Total number of compounds	Selection after Lipinski’s rule	Format	Retrieval date
Natural Product library [17]	3948	203	3D-SDF	25 May 2023
MPD3 medicinal plant database [18]	2295	1716	3D-SDF	27 May 2023
Alkaloid compound library [19]	407	314	3D-SDF	14 June 2023
Flavonoid compound library [20]	241	229	3D-SDF	14 June 2023
Enamine antiviral library [15]	3200	3200	3D-SDF	14 June 2023
Express-ick library [19]	3010	2902	3D-SDF	14 June 2023
Total compounds	13101	8564		

### Protein retrieval and preparation

The X-ray crystal structure of the DPol (PDB ID: 8HG1, Chain A) and A42R (PDB ID: 4QWO) proteins were retrieved from the Protein Data Bank. We selected to target these MPXV proteins based on previously reported studies that identified these to be favorable druggable targets [22,23]. The chosen proteins structures underwent further processing using the PyMOL molecular graphics system (Schrödinger, LLC, v2.0) [24] which involved the removal of redundant chains and DNA molecules. Subsequently, the proteins were imported into the Molecular Operating Environment (MOE) 2022.02 software for preparation [25]. This preparation included steps such as removing water molecules, adding hydrogen atoms, and assigning charges using the OPLS-AA molecular mechanics force field. Previous studies have used a ligand bound to DPol as the active site [26]. We used that, along with the tope site identified by MOE built-in site finder combination, as an active site. For A42R, we used the previously reported active sites that were also identified by the MOE site finder as the tope predicted active site [22].

### Molecular docking

The chosen proteins structures underwent further processing using the PyMOL molecular graphics system (Schrödinger, LLC, v2.0) [24] which involved the removal of redundant chains and DNA molecules. Subsequently, the proteins were imported into the Molecular Operating Environment (MOE) 2022.02 software for preparation [25]. This preparation included steps such as removing water molecules, adding hydrogen atoms, and assigning charges using the OPLS-AA molecular mechanics force field. Previous studies have used ligand bound to DPol as active site [26], which we used along with the tope site identified by MOE built-in site finder combination as an active site. For A42R, we used the previously reported active sites that were also identified by the MOE site finder as the tope predicted active site [22]. Docking parameters used: i. placement: triangle matcher; ii. refinement: induced fit; iii. Score: London dG scoring to estimates the free energy of binding of the ligand from a given pose; iv. GBVI/WSA ΔG [Corbeil]: forcefield-based scoring function to estimates the free energy of binding of the ligand from a given pose; v. total number of poses:30 with 5 poses for further refinement.

### Molecular dynamics (MD) simulation

To gain further insights into the stability and interaction dynamics of the top ten docked complexes (two from each library of focus), MD simulation was initiated. This entire process was carried out using Schrödinger’s Desmond software [27]. As previously discussed [28], with few changes, the energy of the entire system was minimized using the OPLS3e force field. A TIP3P water solvent model was applied around the complexes within a 10 Å orthorhombic box. Following ion addition and system neutralization, the system underwent reevaluation. The simulation extended over 20 ns, encompassing 1000 frames, with temperature equilibration set at 300 K and pressure maintained at 1 bar. To ensure system relaxation after minimization, the production phase spanned 20 ns. Output data, including root-mean-square deviation (RMSD), root-mean-square fluctuation (RMSF) and ligand properties were subsequently visualized and analyzed using the Desmond package’s Simulation Interaction Diagram (SID) tool. The RMSD is used to measure the average change in displacement of a selection of atoms for a particular frame with respect to a reference frame. It is calculated for all frames in the trajectory. The RMSD and RMSF was calculated for Cα atoms of the protein, with RMSD for frame *x*:


$$RMSD_{x}=\;\sqrt{\frac{1}{N}\sum_{\;i\;=1}^{N\;}\left(r_{i}^{\prime }(t_{x})\right)-\;r_{i}(t_{ref}){)}^{2}\;}$$

where *N* is the number of atoms in the atom selection; *t*_*ref*_ is the reference time, (typically the first frame is used as the reference and it is regarded as time t = 0); and *r’* is the position of the selected atoms in frame *x* after superimposing on the reference frame, where frame *x* is recorded at time *t*_*x*_. The procedure is repeated for every frame in the simulation trajectory.

RMSF is useful for characterizing local changes along the protein chain. The RMSF for residue *I* was calculated as:


$$RMSF_{i}=\;\sqrt{\frac{1}{T}\sum_{\;t\;=1}^{T\;}<\left(r_{i}^{\prime }(t)\right)-\;r_{i}\left(t_{ref}\right){)}^{2}\;>}$$

where *T* is the trajectory time over which the RMSF is calculated, *t*_*ref*_ is the reference time, *r*_*i*_ is the position of residue *i; r’* is the position of atoms in residue *i* after superposition on the reference, and the angle brackets indicate that the average of the square distance is taken over the selection of atoms in the residue.

### ADMET analysis

Finally, we performed the absorption, distribution, metabolism, excretion, and toxicity (ADMET) analysis of the selected top hit compounds and compared it to reported ligands. ADMET parameters are critical factors in drug discovery and development. An effective drug candidate must not only demonstrate strong efficacy against the intended therapeutic target but also exhibit suitable ADMET characteristics at therapeutic doses. We first converted the ligands in SDF format to Simplified Molecular Input Line Entry System (SMILE) [29] followed by ADMET analysis using the swissADME and pkCSM online tool [30].

### Statistical analysis

Statistical analysis was performed using the GraphPad Prism (version 10.4.2). For molecular docking analysis, One-Way ANOVA followed by Dunnet posthoc test and data are presented as the mean ± SEM of triplicate. For molecular dynamic simulations, repeated measures One-Way ANOVA followed by Dunnet posthoc text was used and the data are presented as the mean ± SEM of duplicates as previously reported [31]. (significance: ns = non-significant; * = *p* ≤ 0.05; ** = *p* ≤ 0.01; *** = *p* ≤ 0.001; **** = *p* ≤ 0.0001).

## Results

### Molecular docking of DNA polymerase

**Fig 2:**
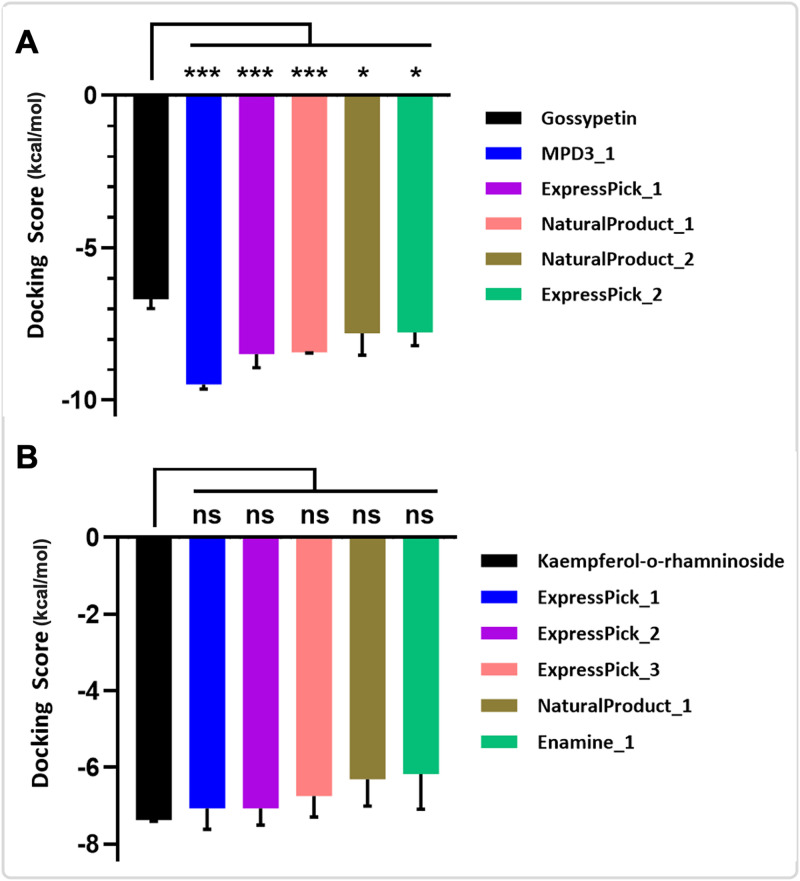
(A and B) Molecular docking score analysis of 3 replicates followed by statistical analysis performed using GraphPad Prism (version 10.4.2). One way ANOVA followed by Dunnet posthoc test was used. Significance: ns = p > 0.05, * = *p* ≤ 0.05, ** = *p* ≤ 0.01, *** = *p* ≤ 0.001, **** = *p* ≤ 0.0001.

In the realm of pharmaceutical design, molecular docking studies serve as an invaluable approach for comprehending the interactions between ligands and proteins. Molecular docking, a simulation technique of particular efficacy, employs energy minimization and binding energy calculations to elucidate the interactions between drugs and their target proteins [32]. Herein, we employed DPol as the target protein, subjecting it to molecular docking with Gossypetin (reference compound) and various other compounds sourced from six distinct libraries, namely the NaturalProduct library, MPD3 medicinal plant database, Alkaloid library, Flavonoid library, Enamine antiviral library, and Express-pick library (Fig 2 and Table 1). All of the top 5 hit compounds displayed significantly lower docking scores (DPol-MPD3_1: -9.48kcal/mol, DPol-ExpressPick_1: -8.49kcal/mol, DPol - NaturalProduct_1-8.43kcal/mol:, DPol-NaturalProduct_2: -7.81kcal/mol and DPol-ExpressPick_2: -7.77kcal/mol) as compared to the reference (DPol-Gossypetin:-6.68kcal/mol). These docking score indicate better binding of ligands to the DPol (Fig 2 and Table 1).

**Fig 3:**
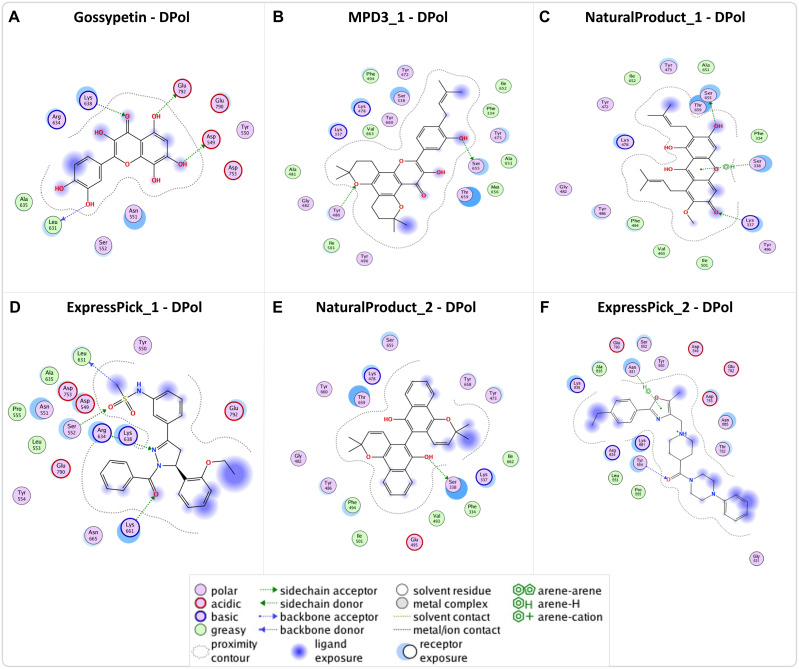
2D structures and the binding pockets of compounds in Dpol protein: (A-D) 2D structure of the ligands in the binding pocket of DPol, illustrating molecular interactions between each ligand’s atoms and amino acid residues. The types of interactions (hydrogen, ionic, arene) and details regarding ligand exposure, acceptor/donor, polarity, acidic/basic, and greasy or neutral residues of the ligand are presented.

**Fig 4:**
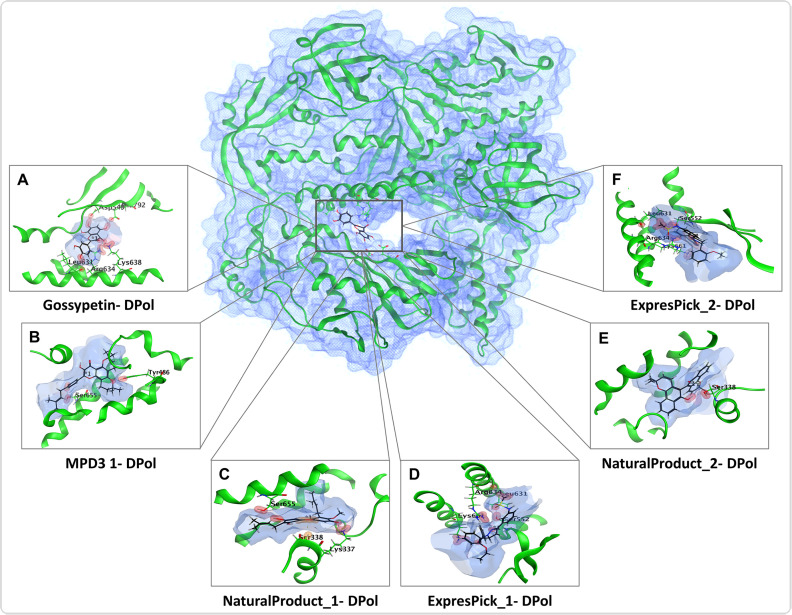
3D structure of DPol and bound ligands in the binding pockets: (A-D) three-dimensional (3D) representation of the ligands in the binding pocket of DPol, depicting the 3D orientation of the ligands, the identities of interacting protein residues, and the ligand’s atoms.

Following the molecular docking analysis, five hits revealed various interactions, including hydrogen bonds and aromatic interactions with DPol (Figs 3 and 4 and Table 2). DPol-MPD3_1 formed two hydrogen bonds with the Tyr486 and Ser655 residues of the receptor, while DPol-Express Pic_1 engaged in four hydrogen bonds with the Ser552, Leu631, Arg634, and Lys661 residues. DPol - NaturalProduct_1 have shown two hydrogen bonds with the Lys337 and Ser655 residues and one arene bond (cyclic aromatic ring of ligand forming bond with amino acid residues of the protein) with the Ser338 whereas DPol-NaturalProduct_2 exhibited interaction through hydrogen bond with the Ser338 residue. DPol-ExpressPick_2 demonstrated four hydrogen bonds with the Ser552, Leu631, Arg634, and Lys661 residues. Overall, all these hit compounds have shown significantly lower docking score as compared to the already reported Gossypetin reference (standard), indicating the potential of these compounds to be favorable drug candidates.

**Table 2:** Molecular docking scores, a list of predicted interacting residues, and types of interactions from DPol and A42R proteins of MPXV with the top ten hits.

Docked complex	Docking scores (kcal/mol) ± SEM	H bonds	Ionic Bonds	Arene Bonds	Total Interactions
DPol-Gossypetin (Reference)	-6.68 ± 0.17	Asp549, Leu631, Arg634, Lys638, Lys638, Glu792	–	–	6
MPD3_1	-9.48 ± 0.09	Tyr486, Ser655	–	–	2
DPol-NaturalProduct_1	-7.81 ± 0.41	Lys337, Ser655	–	Ser338	3
DPol-ExpressPick_1	-8.49 ± 0.25	Ser552, Leu631, Arg634, Lys661	–	–	4
DPol-NaturalProduct 2	-8.43 ± 0.01	Ser338	–	–	1
DPol-ExpressPick_2	-7.77 ± 0.25	Ser552, Leu631, Arg634, Lys661	–	–	4
A42R-Kaempferol 3-O-rhamninoside (Reference)	-7.37 ± 0.02	Met1, Met1, Met1, Ala2, Trp4	–	–	5
A42R-ExpressPick_1	-7.07 ± 0.32	Glu3, Arg127	–	His124	3
A42R-ExpressPick_2	-7.07 ± 0.21	Met1	–	–	1
A42R-ExpressPick_3	-6.75 ± 0.26	Glu3, Arg127	Glu3	His124	4
A42R-NaturalProduct_1	-6.31 ± 0.33	–	–	Trp4	1
DPol-Gossypetin (Reference)	-6.18 ± 0.43	Met1, His124, Arg127	–	Trp4	4

### Molecular docking of profilin-like protein A42R

**Fig 5:**
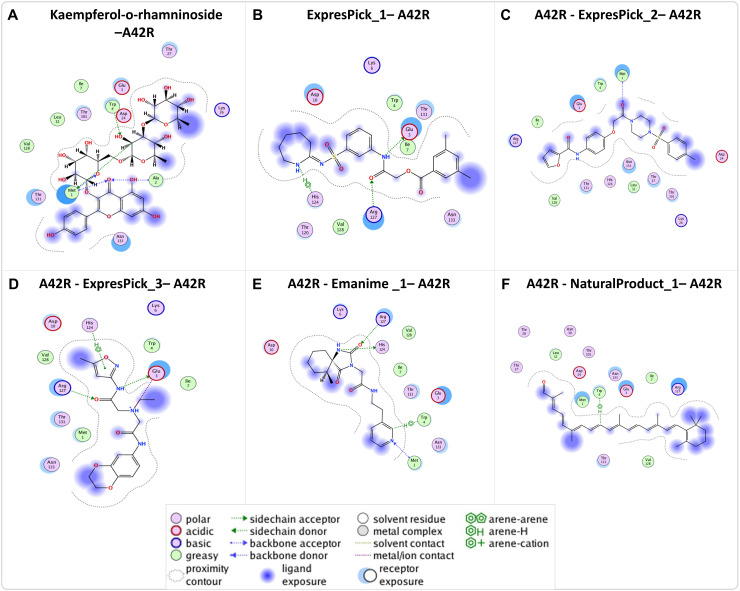
2D structures and the binding pockets of compounds in A42R protein: (A-D) The molecular interactions between the atoms of each ligand and the amino acid residues in the binding pocket of A42R are depicted in the 2D structure of the ligands. The types of interactions (ionic, hydrogen, and arene) as well as information on the polarity, acceptor/donor, ligand exposure, acidic/basic, and greasy or neutral resides of the ligand are clarified.

**Fig 6:**
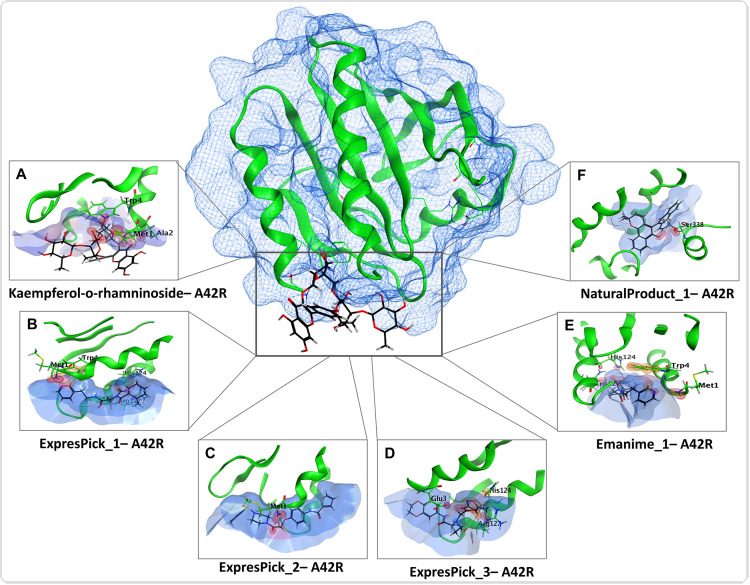
3D structure of DPol and bound ligands in the binding pockets: (A-D) Three-dimensional illustration of the ligand in the binding pocket of A42R, showing the atoms of the ligand, the corresponding protein residues that interact with it, and its orientation in three dimensions.

After performing molecular docking of A42R protein of MPXV with six different compound libraries, along with Kaempferol 3-O-rhamninoside (reference compound), many compounds showed similar docking scores to the reference compound (Figs 5 and 6 and Table 2). Among these compounds, A42R-ExpressPick_1 and A42R-ExpressPick_2 have the docking scores of -7.07kcal/mol. In addition to these, A42R-ExpressPick_3, A42R-NaturalProduct_1 and A42R-Enamine_1 displayed docking scores of -6.75kcal/mol, -6.31kcal/mol and -6.18kcal/mol respectively. Statistical analysis revealed no significant differences between the top 5 hit compounds identified in this study and the reference compound.

The selected top five compounds showed significant interactions with A42R protein, these interactions were arene and H-bonds, as detailed in Figs 5 and 6 and Table 2. A42R-ExpressPick_1 formed two H-bonds with the Glu3 and Arg127 residues and one arene bond with His124 residue. Whereas A42R-ExpressPick_2 interacted with the Met1 residue though H-bond, A42R-ExpressPick_3 on the other hand displayed two H-bonds with the Glu3, Arg127 residues, one ionic interaction with Glu3, and one arene bond with the His124 residue. A42R-NaturalProduct_1 interacted through an H-bond with the Trp4 residue of A42R, while 42R-Enamine_1 demonstrated three H-bonds with the Met1, His124, and Arg127 residues, and one arene bond with the Trp4 residue. All the hit compounds have shown similar docking score as compared to the already reported reference(standard) Kaempferol 3-O-rhamninoside.

### Molecular dynamic simulations of DPol and A42R with selected ligands

Molecular dynamics (MD) simulations represent a widely utilized technique for assessing atomic behavior, structural stability, and atomic-level conformational changes [33]. In this study, the top-ranking hits from the NaturalProduct library, MPD3 medicinal plant database, alkaloid library, flavonoid library, Enamine antiviral library, and express-pick library underwent MD simulations using Schrodinger’s Desmond software (v. 2022–1).

#### Root mean square deviation (RMSD).

**Fig 7:**
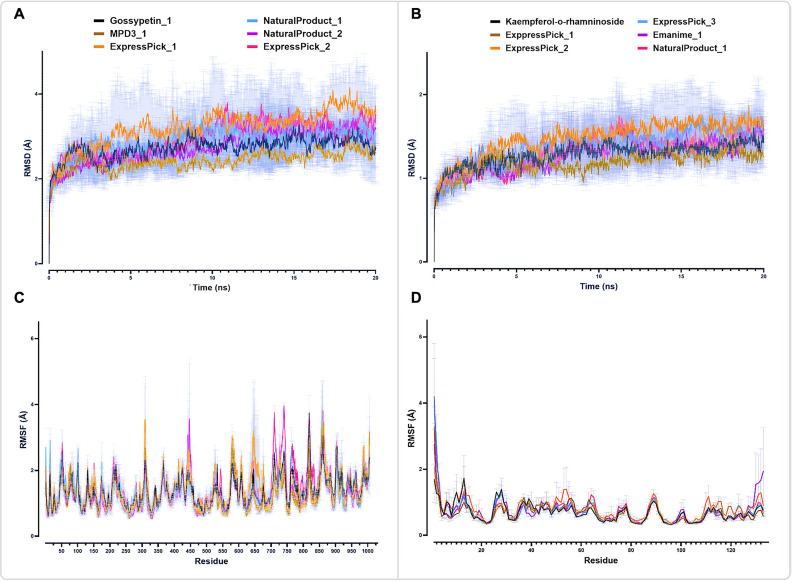
Root mean square deviation (RMSD) and root mean square fluctuation (RMSD): (A and B) The RMSD values of the MPXV’s A42R and DPol proteins (Cα atoms) backbone are shown bound to hit compounds during the molecular dynamics (MD) simulation. (C and D) Illustration of the RMSF values of the MPXV’s A42R and DPol proteins backbone bound to hit compounds throughout the simulation. Statistical analysis was performed using repeated measure One-Way ANOVA followed by Dunnet posthoc test. Mean of each ligand bound proteins complex was compared to the means of the respective reference (standard) bound protein complex throughout the simulation. The data are shown as the mean ± SEM of 2 replicates.

RMSD serves as a prominent index in MD trajectory analysis, offering insights into the stability of conformations [34]. Among the DPol-ligand complexes, DPol-MPD3_1 showed lower RMSD values with an average of 2.40Å as compared to DPol-Gossypetin complex having 2.73Å over the course of the 20 ns MD simulation, indicating a more stable protein-ligand complex (Fig 7A). DPol-NaturalProduct_1 has a similar RMSD value, with an average of 2.75Å. On the other hand, DPol-ExpressPick_1, DPol-ExpressPick_2 and DPol-NaturalProduct_2 have shown higher RMSD values with an average of 3.20Å, 2.90Å, and 2.87Å, respectively. Statistical analysis of the mean of protein-ligand mean over the 20ns simulations revealed that DPol-MPD3_1 has significantly lower RMSD values (*p* ≤ 0.0001) as compared to Gossypetin. DPol-NaturalProduct_2 RMSD was non-significant, while other hit compounds have shown significantly higher RMSD values (*p* ≤ 0.0001) throughout the simulation.

Among all A42R-ligand complexes, A42R-ExpressPick_1 has mean RMSD value of 1.18Å, compared to A42R-Kaempferol 3-O-rhamninoside having 1.28Å, throughout 20ns simulation (Fig 7B). A42R-NaturalProduct_1 have shown a similar RMSD value with a value of 1.27Å. However, A42R-ExpressPick_2, A42R-ExpressPick_3 and A42R-Enamine_1 have shown higher RMSD values with an average of 1.40Å, 1.37Å, and 1.31Å, respectively. Furthermore, the statistical analysis of the mean of protein-ligand MD simulation throughout 20ns has indicated that A42R-ExpressPick_1 has significantly lower RMSD values (*p* ≤ 0.0001), suggesting a more stable protein-ligand complex as compared to A42R-Kaempferol 3-O-rhamninoside. On the other hand, other protein-ligand complexes have shown higher RMSD values (*p* ≤ 0.0001), while A42R-NaturalProduct_1 have non-significant RMSD values compared to that of the A42R-Kaempferol 3-O-rhamninoside.

#### Root mean square fluctuation (RMSF).

RMSF analysis allows for the observation of conformational shifts in residues during the simulation process [4]. The ligand-DPol amino residue fluctuations throughout the 20ns simulation, DPol-ExpressPick_1 and DPol-ExpressPick_2 exhibited higher fluctuations with average RMSF values of 1.45Å and 1.50Å, respectively, as compared to the DPol-Gossypetin (1.36Å) (Fig 7C). DPol-NaturalProduct_1 have shown non-significant RMSF values as compared to A42R-Kaempferol 3-O-rhamninoside. Conversely, DPol-NaturalProduct_2 and DPol-MPD3_1 exhibited lower RMSF values with an average of 1.20Å and 1.24Å, respectively. Statistical analysis indicated that DPol-ExpressPick_1 and DPol-ExpressPick_2 complexes have significantly higher RMSF values showing more flexible protein-ligand complex while DPol-MPD3_1 and DPol-NaturalProduct_2 have significantly lower RMSF values throughout the simulation. DPol-NaturalProduct_1 have shown non-significant difference as compared to of DPol-Gossypetin.

The A42R amino residues’ average RMSF values throughout the 20ns simulations, A42R-ExpressPick_2, A42R-ExpressPick_3, A42R-NaturalProduct_1 and A42R-Enamine_1 showed higher fluctuations as compared to A42R-Kaempferol 3-O-rhamninoside (0.79Å, 0.72Å, 0.73Å, 0.72Å and 0.69Å, respectively) (Fig 7D). On the other hand, A42R-ExpressPick_1 showed lower RMSF values (0.67Å) as compared to A42R-Kaempferol 3-O-rhamninoside. Moreover, A42R-ExpressPick_2 have shown significantly higher RMSF values (*p* ≤ 0.0001) as compared to A42R-Kaempferol 3-O-rhamninoside indicating a more flexible protein-ligand complex. Other protein-ligand complexes RMSF values are statistically non-significant throw-out the simulation time-period.

Molecular dynamics simulations provide crucial insights into the behavior and stability of ligand-protein complexes, shedding light on their dynamic interactions and potential for therapeutic applications. The RMSD and RMSF analyses presented here offer valuable information on the stability and conformational dynamics of the studied complexes, with implications for drug discovery and development.

### Properties of selected ligands

The ligand RMSD indicates its items stability during the MD simulations. The RMSD monitored during MD simulations of 8HG1 and 4QWO proteins bound ligands revealed that NaturalProduct_2 (S1A Fig), and Enamine_1 and ExpressPick_3 (S1F Fig) shows better RMSD as compared to the reference ligands. The compactness of the complexes which primarily influences their rigidity was monitored using the radius of gyration. Higher radius of gyration values indicates greater instability in the complex, while lower values correspond to increased stability [35]. Throughout the simulation period, the radius of gyration values for NaturalProduct_2 shows similar values to that of reference (S1B Fig). On the other hand, enamine_1 and ExpressPick_3 shows better values as compared to the reference ligand (S1G Fig). A probe radius of 1.4 Å, equivalent to the van der Waals surface area of a water molecule, was used to calculate the molecular surface area (MolSA). Throughout the simulation, MolSA values exhibited fluctuations across different ranges. MPD3_1 and ExpressPick_2 and Kaempferol 3-O-rhamninoside show the higher MolSa values bound to DPol and A42R proteins of MPXV, respectively (S1C and S1H Fig). Solvent accessible surface area (SASA) values showed higher values for ExpressPick_1, ExpressPick_2 and NaturalProduct_2 shows better as compared to Gossypetin while Kaempferol 3-O-rhamninoside indicates better SASA scores (S1C and S1I Fig). The polar surface area (PSA) results better scores for both the reference ligands as compared to the selected test ligands (S1E and S1J Fig).

### Pharmacokinetics and physiochemical properties of selected ligands

To distinguish between drug-like and non-drug-like compounds, Lipinski’s rule of five was utilized (Table 3). This rule helps identify a molecule’s drug-like properties based on its structural features [36,37]. Key pharmacokinetic parameters such as hydrogen bond acceptors, hydrogen bond donors, topological polar surface area (TPSA), bioavailability, molecular weight, and consensus log Po/w were assessed using MOE’s built-in descriptor compute tool.

**Table 3:** *In silico* ADMET properties of the selected hit ligands identified in this study.

Ligand-protein complex	Absorption			Distribution		Metabolism		Excretion		Toxicity		
**Water solubility** **Numeric (log mol/L)**	**Caco-2 permeability Numeric (log Papp in 10** ^**–6**^ ** cm/s)**	**Human intestinal absorption (%)**	**VDss (human) Numeric (log mol/L)**	**BBB permeability Numeric (log BB)**	**CYP2D6 substrate (Y/N)**	**CYP12A inhibitor (Y/N)**	**Total clearance** **(ml/min/kg)**	**Renal OCT2 substrate (Y/N)**	**AMES toxicity (Y/N)**	**Skin sensitization (Y/N)**	**Hepatotoxicity (Y/N)**
DPol-Gossypetin (Reference)	-3.065	-0.033	61.155	0.541	-1.538	N	N	-0.17	N	N	N	N
MPD3_1	-3.991	1.245	94.505	0.679	0.015	N	N	-0.411	N	N	N	N
DPol-NaturalProduct_1	-3.736	0.647	0.647	-0.282	-1.154	N	N	0.513	N	N	N	N
DPol-ExpressPick_1	-5.086	1.142	91.986	-0.319	-0.822	N	N	0.741	N	Y	N	Y
DPol-NaturalProduct 2	-3.927	1.133	94.214	-1.251	-0.175	N	N	0.389	N	N	N	Y
DPol-ExpressPick_2	-4.232	1.047	94.186	1.114	0.293	Y	N	0.675	Y	N	N	N
A42R-Kaempferol 3-O-rhamninoside (Reference)	-4.48	-6.63	0.011	0.72	-4.66	Y	Y	9.35	N	N	N	N
A42R-ExpressPick_1	-5.33	0.81	0.81	-0.453	-0.893	N	N	-0.181	N	N	N	N
A42R-ExpressPick_2	-4.448	1.128	90.662	-0.292	-0.763	N	N	0.598	Y	N	N	Y
A42R-ExpressPick_3	-2.761	0.676	73.67	-0.076	-0.922	N	N	0.971	N	N	N	Y
A42R-Enamine_1	-3.654	0.221	67.602	-0.25	-0.876	N	N	1.337	N	N	N	Y
A42R-NaturalProduct_1	-8.113	1.361	0.318	0.318	0.784	N	N	1.368		N		N

ADMET, representing Absorption, Distribution, Metabolism, Excretion, and Toxicity, encompasses crucial pharmacological properties for drug candidates. These properties are essential in drug development, as approximately 50% of drug failures are due to ADMET issues [38]. *In silico* ADMET analyses were conducted using the pkCSM web tool (https://biosig.lab.uq.edu.au/pkcsm/prediction) [39]. For selected ligands, various pharmacokinetic parameters such as water solubility, Caco-2 permeability, human intestinal absorption, blood-brain barrier (BBB) penetration, cytochrome P450 inhibition and substrate status, AMES toxicity, skin sensitization, and hepatotoxicity were evaluated.

Caco-2 permeability: the rate of flux of a compound across polarized Caco-2 cell monolayers; BBB: blood brain barrier; VDss: steady state volume of distribution; CYP2D6: Cytochrome P450 2D6; CYP12A: Cytochrome P450 12A; OCT2: OCT2 is a primarily renal uptake transporter; AMES: carcinogenic effects of ligands on bacterial strain Salmonella typhimurium; Y/N: Yes/N. Cut-off values: Water solubility; the logarithm of aqueous solubility at a temperature of 20–25°C in log mol/L with a cut-off value of −12–2 [40]. Caco-2 cell permeability: a compound is considered to have a high Caco-2 permeability if it has a Papp > 8 x 10–6 cm/s; for intestinal absorption, a molecule with an absorbance of less than 30% is considered to be poorly absorbed; VDss: it is considered low if below 0.71 L/kg (log VDss < -0.15) and high if above 2.81 L/kg (log VDss > 0.45) [41]. BBB permeability: logBB classify compounds as either BBB+ (permeable) or BBB− (non-permeable) with values ranging from −1.00 to +0.63, compounds having a logBB ≥ 0.00 being BBB+ and a logBB of zero implies an equal concentration on both sides of the BBB [42].

As per Lipinski’s Rule of Five criteria in medicinal chemistry, compounds demonstrating druglikeness should possess a molecular weight (M. Weight) not exceeding 500 Da, offer no more than 5 hydrogen bonds (lip_don), accept no more than 10 hydrogen bonds (lip_acc), and exhibit a partition coefficient (h_logP) less than 5 (Table 4) [43]. Non-compliance with these parameters may compromise the compound’s bioavailability. Nonetheless, in therapeutic development, deviations from these guidelines can be addressed through modifications in the administration route or by adding specific moieties to enhance the compound’s bioavailability.

**Table 4:** Physiochemical properties of the top 10 selected hit compounds from 5 libraries.

Compounds	M. Weight	logP	h_acc	h_don	TPSA	Rot_bonds	Lipinski violation
DPol-Gossypetin (Reference)	318.2	0.8	8	5	144.5	1	0
MPD3_1	490.6	4.9	6	2	0.239	3	0
DPol-NaturalProduct_1	410.5	3.37	6	3	152.6	5	0
DPol-ExpressPick_1	463.6	3.11	5	1	96.45	8	0
DPol-NaturalProduct 2	450.5	4.52	4	2	58.92	1	0
DPol-ExpressPick_2	472.6	4.8	4	0	52.82	7	0
A42R-Kaempferol 3-O-rhamninoside (Reference)	740.6	2.57	19	11	308.12	8	3
A42R-ExpressPick_1	457.5	3.16	6	2	122.3	8	0
A42R-ExpressPick_2	483.5	3.34	7	1	117	9	0
A42R-ExpressPick_3	375.4	2.45	6	3	107.13	9	0
A42R-Enamine_1	344.4	2.39	4	2	91.4	6	0
A42R-NaturalProduct_1	416.64	7.5	1	0	17.07	9	0

h_logP: higher logP (partition coefficient between organic and aqueous phases); h_acc: hydrogen bond acceptor; h_don: hydrogen bond donor; TPSA: Topological surface area, Rot_bonds: rotatable bonds.

**Fig 8:**
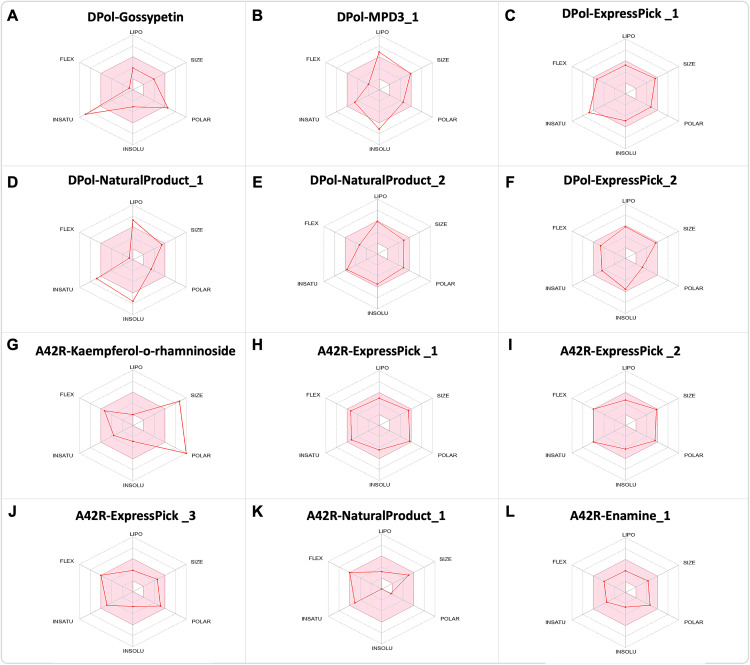
Oral bioavailability of the hit compounds: (A-L) The color zone represents the physiochemical space for oral bioavailability (retrieved from swissADME). LIPO (lipophilicity): -0.7 < XLOGP3<+5.0; SIZE: < 500g/mol; POLAR (Polarity): 20Å^2 ^< TPSA<130Å^2^; INSOLU (Insolubility) -6 < LogS (ESOL) < 0; INSATU (Instauration): 0.25 < fraction Csp3 < 1; FLEX (Flexibility): 0 < Num. rotatable bonds <9.

Furthermore, we checked the swissADME [30] online database for oral bioavailibity of these compounds against the reference ligands (Gossypetin and Kaempferol 3-O-rhamninoside). Overall, most of the selected ligands show better oral bioavailability than the already reported ligands (Fig 8A–L).

Finally, we saved the selected ligands as SDF format using MOE and then converted to Simplified Molecular Input Line Entry System (SMILES) using the online tool smiles translator and structure file generator [29]. Then the SMILES were used to retrieve the details of each ligands from PubChem [44], such as their names, IDs and formulae (Table 5). The IUPAC names of the ligands are provided in the S1 Text file.

**Table 5:** Identification of the hit compounds in the docked complexes, PubChem IDs and chemical formula.

Docked Complex	Pubchem IDs	Formula	Molecular Weight g/mol
DPol-Gossypetin (Reference)	5280647	C_15_H_10_O_8_	318.23
MPD3_1	25178185	C_30_H_34_O_6_	490.6
DPol-NaturalProduct_1	5281650	C_24_H_26_O_6_	410.5
DPol-ExpressPick_1	5233936	C_25_H_25_N_3_O_4_S	463.6
DPol-NaturalProduct 2	161453	C_30_H_26_O_4_	450.5
DPol-ExpressPick_2	3242611	C_29_H_36_N_4_O_2_	472.6
A42R-Kaempferol 3-O-rhamninoside (Reference)	14186901	C_33_H_40_O_19_	740.7
A42R-ExpressPick_1	5204354	C_23_H_27_N_3_O_5_S	457.5
A42R-ExpressPick_2	4870002	C_24_H_25_N_3_O_6_S	483.5
A42R-ExpressPick_3	2114093	C_18_H_23_N_4_O_5_^+^	375.4
A42R-Enamine_1	46546402	C_18_H_24_N_4_O_3_	344.4
A42R-NaturalProduct_1	5478003	C_30_H_40_O	416.6

## Discussion

Both humans and animals can contract monkeypox, a viral illness caused by MPXV. MPXV requires a specialized DNA polymerase (DPol) enzyme for replication and dissemination within the host. This enzyme plays a pivotal role in manipulating the host’s immune response, contributing to the development of the monkeypox infection [45,46]. Consequently, comprehending the structure and function of DPol is of principal importance for the development of effective antiviral treatments and vaccines to combat MPXV detrimental effects [46].

While there are available treatments such as the JYNNEOS vaccine, tecovirimat, brincidofovir, and cidofovir for monkeypox, the exploration of adjuvant therapies utilizing phytochemicals and immune-enhancing diets holds promise in countering the spread of MPXV. Many phytochemicals have shown potential in reducing viral replication and bolstering host defenses, making them intriguing candidates for addressing viral diseases like influenza, HIV, herpes simplex virus, and SARS-CoV-2 [47,48]. Although promising in preclinical research, the practical application of phytochemicals as antiviral medications faces challenges due to the cost of isolation and production, the need for extensive clinical investigations to determine optimal dosages and delivery methods, and the imperative for safety and efficacy [48,49].

In our study, we employed virtual screening techniques on various compound libraries, including the NaturalProduct library, MPD3 medicinal plant database, Alkaloid library, Flavonoid library, Enamine antiviral library, and Express-pick library (Table 1), to identify compounds capable of inhibiting DPol and A42R activities in MPXV. The docking analysis unveiled several compounds having notable interactions with DPol including DPol-MPD3_1, DPol-NaturalProduct_1

, DPol-NaturalProduct_2, DPol-ExpressPick_2. These compounds exhibited an average docking score of -9.48kcal/mol, -7.81kcal/mol, -8.49kcal/mol, -8.43kcal/mol and -7.77kcal/mol, respectively (Table 2). Similarly, docking analysis identified top 5 hit compounds that interact with A42R protein of MPXV, including A42R - ExpressPick_1, A42R - ExpressPick_2, A42R - ExpressPick_3, A42R - NaturalProduct_1 and A42R - Emanime_1 with an average docking scores of -7.37kcal/mol, -7.06kcal/mol, -7.07kcal/mol, -6.75kcal/mol, -6.31kcal/mol and -6.18kcal/mol, respectively. Notably, these compounds displayed substantially lower docking scores compared to FDA-approved drugs for MPXV, namely, cidofovir and bricindofovir. A previous study has reported Gossypetin (-6.3) to have similar lower score than cidofovir (-6.0) and bricindofovir (-5.1), respectively, in complexes with the DPol of MPXV [26]. Thus, we used Gossypetin as a reference/standard compound for molecular docking in this study.

Furthermore, our molecular docking analysis identified residues Lys337, Ser338, Tyr486, Asp549, Ser552, Leu631, Arg634, Lys638, Ser655, Lys661 and Glu792 making H bonds with ligands (Figs 3 and 4 and Table 2). Ser338 also makes an arene (aromatic bonds between ligand and amino acid residue of protein) with DPol-NaturalProduct_1.On the other hand, A42R H bond making residues include Met1, Ala2, Glu3, Trp4, His124 and Arg127 (Figs 5 and 6, and Table 2). Glu3 also makes an ionic bond with ExpressPick_3- A42R, while Trp4 and His124 makes arene bonds with ExpressPick_1- A42R, ExpressPick_3- A42R, NaturalProduct_1- A42R and Enamine_1- A42R.

Subsequent MD simulations highlighted the structural stability of the DPol and A42R protein-ligand complexes over a 20ns trajectory. Among the DPol complexes, DPol-MPD3_1 demonstrated the lowest RMSD values with an average of 2.40Å which was notably lower than that of the DPol-Gossypetin (2.73Å), while DPol-NaturalProduct_1 (2.75Å) showed non-significant RMSD values indicating good stabilities (Fig 7A). On the other hand, DPol-ExpressPick_1, DPol-ExpressPick_2 and DPol-NaturalProduct_2 revealed higher RMSD values of 3.20Å, 2.90Å, and 2.87Å, respectively, suggesting comparatively less stable interactions. Statistical analysis suggested that DPol-MPD3_1 had significantly lower RMSD values (*p* ≤ 0.0001) as compared to Gossypetin (reference), while RMSD differences for NaturalProduct_2 was non-significant. Likewise, in the A42R-ligand complexes, A42R-ExpressPick_1 showed the most stable interaction profile with a mean RMSD of 1.18 Å throughout the simulation time-period as compared to A42R-Kaempferol 3-O-rhamninoside (1.28 Å). A42R-NaturalProduct_1 exhibited a similar RMSD of 1.27 Å (Fig 7B). Other ligands, including A42R-ExpressPick_2, ExpressPick_3, and Enamine_1, demonstrated slightly elevated RMSD values (1.4Å, 1.37Å, and 1.31Å, respectively). Statistical analysis revealed that A42R-ExpressPick_1 has a significantly more stable complex (*p* ≤ 0.0001), while NaturalProduct_1 remained non-significant. RMSF analyses further provided insights into the flexibility of key residues within these complexes. With DPol-ligand complexes, ExpressPick_1 and ExpressPick_2 displayed higher RMSF fluctuations (1.45Å and 1.50Å) suggesting a more flexible protein-ligand complex. In contrast to DPol-Gossypetin (1.36Å), while NaturalProduct_2 and MPD3_1 exhibited lower RMSF values (1.20Å and 1.24Å) indicating a more rigid complex (Fig 7C). RMSF value for NaturalProduct_1 was similar to that of the reference DPol-Gossypetin. Similarly, for A42R complexes, ExpressPick_2 (0.79Å), ExpressPick_3 (0.72Å), NaturalProduct_1 (0.73 Å), and Enamine_1 (0.72 Å) showed higher flexibility as compared to Kaempferol 3-O-rhamninoside (0.69 Å). On the other hand, ExpressPick_1 demonstrated a lower RMSF of 0.67 Å (Fig 7D). Among these, only A42R-ExpressPick_2 showed statistically significant RMSF increases (*p* ≤ 0.0001, Mean was calculated for the whole data points calculated throughout the 20ns simulation), showing a more flexible protein-ligand complex, whereas the rest were non-significant. Collectively, this MD analysis provides a better stability of MPD3_1 with DPol and ExpressPick_1 with A42R, suggesting their potentials for further therapeutic evaluation.

Furthermore, we evaluated the ADMET, and physiological properties of our hit compounds, revealing promising results as compared to the already reported ligands (Fig 7 and Table 4). Finally, the oral bioavailability of these compounds also shows better results in most cases, except for naturalproduct_2 ligand bound to DPol.

Computational methods are useful for identifying potential antiviral compounds, but they have their limitations. Since these predictions are based purely on computational models, they can sometimes produce false positives which means that a compound might seem effective in theory but may not be as effective *in vitro* and *in vivo*. To examine the antiviral effect of these inhibitors *in vitro*, the plaque reduction assay (PRA), where plaque number reduction indicates antiviral effects, can be performed [50]. Cytopathic effect (CPE) inhibition assays will help in evaluating how well compounds can protect host cells from viral infection. CPE can be combined with viability assays like MTT assay (3-(4,5-dimethylthiazol-2-yl)-2,5-diphenyltetrazolium bromide) [51,52]. The virus yield reduction assay may be used to measure the decline in viral population in the presence of inhibitors, through quantitative PCR (qPCR) analysis can also be used to evaluate viral titers or genome copies can be utilized. Immunofluorescence and ELISA assays to detect viral proteins in infected cells with specific antibodies, providing insights into how inhibitors impact viral replication or assembly [53,54]. An additional challenge is that these findings have not been tested in lab experiments or clinical settings yet, so we do not know for sure whether they are safe or effective. To confirm their potential, further laboratory and animal studies are essential before considering them for real-world treatments.

## Conclusion

The protein-ligand molecular docking results have unveiled ten noteworthy medicinal compounds: Dorsilurin K, Mangostin against DPol, and [2-oxo-2-[3-(3,4,5,6-tetrahydro-2H-azepin-7-ylsulfamoyl)anilino]ethyl] 3,5-dimethylbenzoate and N-[4-[2-[4-(4-methylphenyl)sulfonylpiperazin-1-yl]-2-oxoethoxy]phenyl]furan-2-carboxamide against A42R are the top two candidates for each protein. This molecular docking investigation has highlighted their high-affinity interactions with DPol and A42R, suggesting their potential to inhibit the replication of MPXV. Furthermore, the MD simulations have affirmed the stability of these lead compounds, exhibiting minimal deviations throughout the simulation period. Additionally, ADMET analysis revealed favorable pharmacokinetic properties for these lead compounds, indicating their potential for oral bioavailability and therapeutic application. Given these interesting findings, future research should focus on *in vitro* and *in vivo* validation to assess the efficacy (such as PRA, CPE, MTT, RT-qPCR and ELISA assays), toxicity, and optimal dosage of these compounds. Finally, structural modifications and formulation improvements could enhance bioavailability and therapeutic action, paving the way for the development of novel antiviral treatments against MPXV.

### Ethical statement

There are no human/animal subjects involved; thus, no ethical approval was required for this study.

## Supporting information

S1 Fig Properties of ligands during the MD simulations. (A and B) RMSD of ligands atoms, (C and D) radius of gyration, (E and F) molecular surface area (MolSA), (G and H) Solvent accessible surface area (SASA), (I and J) polar surface area (PSA).
(TIFF)

S1 Text IUPAC names of the top 10 hit ligands. (DOCX)

S1 Data Including: ExcelFile_Sheet_1: Molecular docking score Means and SEM used, and triplicate data points used, for Fig 1 and Table 2. One way ANOVA followed by Dunnet posthoc test made via using GraphPad Prism 10.4.2. ExcelFile_Sheet_2: Molecular Docking contacts DPol and ligands showing type of bond, chain of the protein used and its interacting residues, Energy, (interaction energy in kj/mol), Dist (distance between interaction, Area (the area on the contact surface), Atom A and B (the atoms in the residue and ligands involved in the contact, respectively). ExcelFile_Sheet_3: Molecular Docking contacts DPol and ligands showing type of bond, chain of the protein used and its interacting residues, Energy, (interaction energy in kj/mol), Dist (distance between interaction, Area (the area on the contact surface), Atom A and B (the atoms in the residue and ligands involved in the contact, respectively). ExcelFile_Sheet_4: RMSD Values calculated during Molecular Dynamic Simulation (MDS) over the period of 20ns for the DPol_ligand complexes. ExcelFile_Sheet_5: RMSD Values calculated during Molecular Dynamic Simulation (MDS) over the period of 20ns for the A42R_ligand complexes. ExcelFile_Sheet_6: RMSF values calculated during Molecular Dynamic Simulation (MDS) over the period of 20ns for the DPol_ligand complexes. ExcelFile_Sheet_7: RMSF values calculated during Molecular Dynamic Simulation (MDS) over the period of 20ns for the A42R_ligand complexes. ExcelFile_Sheet_8: DPol bound ligands properties. ExcelFile_Sheet_9: A42R bound ligands properties.
(XLSX)
