# Supplementary figures and images for: Computational screening of antiviral candidates for Monkeypox virus DNA polymerase and A42R protein
Source: PLoS Negl Trop Dis. 2025 Jul 29;19(7):e0013312. doi: 10.1371/journal.pntd.0013312 (PMC12393763; doi:10.1371/journal.pntd.0013312)

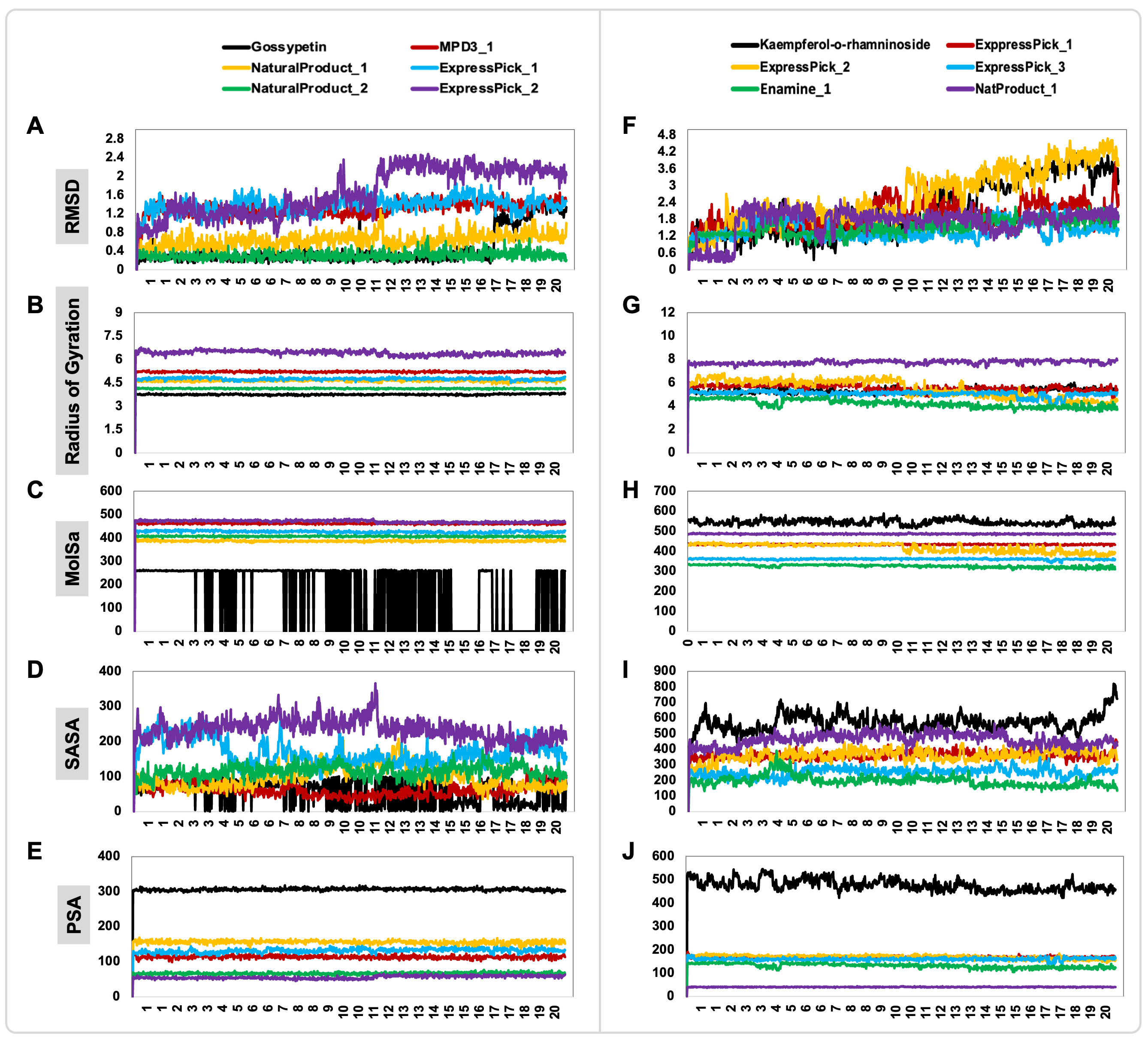

Supplement: S1 Fig — (A and B) RMSD of ligands atoms, (C and D) radius of gyration, (E and F) molecular surface area (MolSA), (G and H) Solvent accessible surface area (SASA), (I and J) polar surface area (PSA). (TIFF) [file pntd.0013312.s001.tiff]
