# Supplementary material for: Computational screening of antiviral candidates for Monkeypox virus DNA polymerase and A42R protein
Source: PLoS Negl Trop Dis. 2025 Jul 29;19(7):e0013312. doi: 10.1371/journal.pntd.0013312 (PMC12393763; doi:10.1371/journal.pntd.0013312)
Supplement: S1 Text — (DOCX) [file pntd.0013312.s002.docx]

**S1 Text:** Protein-Ligand complex and the IUPAC names identified in this study as potential MPXV inhibitors.

1. **DPol-Gossypetin (Reference):** 2-(3,4-Dihydroxyphenyl)-3,5,7,8-tetrahydroxy-2,3-dihydrochromen-4-one
2. **DPol-MPD3_1:** 5-hydroxy-4-[3-hydroxy-4-(3-methylbut-2-enyl)phenyl]-10,10,16,16-tetramethyl-3,9,15-trioxatetracyclo[12.4.0.02,7.08,13]octadeca-1,4,7,13-tetraen-6-one Synonyms: Mangostin.
3. **DPol-NaturalProduct_1:** 1,3,6-trihydroxy-7-methoxy-2,8-bis(3-methylbut-2-enyl)xanthen-9-one.

Synonyms: Dorsilurin K

1. **DPol-ExpressPick_1:** N-[3-[2-benzoyl-3-(2-methoxyphenyl)-3,4-dihydropyrazol-5-yl]phenyl]methanesulfonamide.
2. **DPol-NaturalProduct 2:** 5-(6-hydroxy-2,2-dimethylbenzo[h]chromen-5-yl)-2,2-dimethylbenzo[h]chromen-6-ol.

Synonyms: Tectol

1. **DPol-ExpressPick_2:** [1-[[2-(4-ethylphenyl)-5-methyl-1,3-oxazol-4-yl]methyl]piperidin-4-yl]-(4-phenylpiperazin-1-yl)methanone.
2. **A42R-Kaempferol 3-O-rhamninoside (Reference):** 3-[6-[[3,5-dihydroxy-6-methyl-4-(3,4,5-trihydroxy-6-methyloxan-2-yl)oxyoxan-2-yl]oxymethyl]-3,4,5-trihydroxyoxan-2-yl]oxy-5,7-dihydroxy-2-(4-hydroxyphenyl)chromen-4-one
3. **A42R-ExpressPick_1:** [2-oxo-2-[3-(3,4,5,6-tetrahydro-2H-azepin-7-ylsulfamoyl)anilino]ethyl] 3,5-dimethylbenzoate.
4. **A42R-ExpressPick_2:** N-[4-[2-[4-(4-methylphenyl)sulfonylpiperazin-1-yl]-2-oxoethoxy]phenyl]furan-2-carboxamide.
5. **A42R-ExpressPick_3:** [2-(2,3-dihydro-1,4-benzodioxin-6-ylamino)-2-oxoethyl]-ethyl-[2-[(5-methyl-1,2-oxazol-3-yl)amino]-2-oxoethyl]azanium.
6. **A42R-Enamine_1:** 2-(6-methyl-2,4-dioxo-1,3-diazaspiro[4.5]decan-3-yl)-N-(2-pyridin-3-ylethyl)acetamide.
7. **A42R-NaturalProduct_1:** (2E,4E,6E,8E,10E,12E,14E,16E)-2,6,11,15-tetramethyl-17-(2,6,6-trimethylcyclohexen-1-yl)heptadeca-2,4,6,8,10,12,14,16-octaenal
   Synonyms: Apocarotenal
